# A Novel Feature Selection with Hybrid Deep Learning Based Heart Disease Detection and Classification in the e-Healthcare Environment

**DOI:** 10.1155/2022/1167494

**Published:** 2022-09-28

**Authors:** Dwarakanath B., Latha M., Annamalai R., Jagadish S. Kallimani, Ranjan Walia, Birhanu Belete

**Affiliations:** ^1^Department of Information Technology, SRM Institute of Science and Technology, Ramapuram, Chennai, India; ^2^Department of Artificial Intelligence, Amrita School of Engineering, Amrita Vishwa Vidyapeetham, Chennai, India; ^3^Department of Artificial Intelligence and Machine Learning, M S Ramaiah Institute of Technology, Bangalore, India; ^4^Department of Electrical Engineering, Model Institute of Engineering and Technology, Jammu, Jammu and Kashmir, India; ^5^School of Electrical and Computer Science Engineering, Jimma Institute of Technology, Jimma, Ethiopia

## Abstract

With the advancements in data mining, wearables, and cloud computing, online disease diagnosis services have been widely employed in the e-healthcare environment and improved the quality of the services. The e-healthcare services help to reduce the death rate by the earlier identification of the diseases. Simultaneously, heart disease (HD) is a deadly disorder, and patient survival depends on early diagnosis of HD. Early HD diagnosis and categorization play a key role in the analysis of clinical data. In the context of e-healthcare, we provide a novel feature selection with hybrid deep learning-based heart disease detection and classification (FSHDL-HDDC) model. The two primary preprocessing processes of the FSHDL-HDDC approach are data normalisation and the replacement of missing values. The FSHDL-HDDC method also necessitates the development of a feature selection method based on the elite opposition-based squirrel searchalgorithm (EO-SSA) in order to determine the optimal subset of features. Moreover, an attention-based convolutional neural network (ACNN) with long short-term memory (LSTM), called (ACNN-LSTM) model, is utilized for the detection of HD by using medical data. An extensive experimental study is performed to ensure the improved classification performance of the FSHDL-HDDC technique. A detailed comparison study reported the betterment of the FSHDL-HDDC method on existing techniques interms of different performance measures. The suggested system, the FSHDL-HDDC, has reached its maximum level of accuracy, which is 0.9772.

## 1. Introduction

E-healthcare could give medical diagnosis services anytime and anyplace, gaining interest recently. Online medical diagnosis is convenient and adaptable since it reduces the wait time to see a doctor and breaks geographical restrictions [1]. E-healthcare models use data mining (DM) to forecast hidden diseases from medical data. With obtained medical data, healthcare centres can develop detection systems by using medical data mining and employing skyline queries to assess patients' health conditions quickly and provide online medical diagnosis services [2]. In typical online medical systems, healthcare information data are normally maintained in discrete healthcare centres, and an individual healthcare centre collects only a narrower collection of healthcare information [3].

Internet of things (IoT) expansion and clinical trials have improved remote healthcare monitoring [4]. IoT connects physical items to continuously monitor physical occurrences. With IoT, heart disease monitoring systems may capture and send patients' physical parameters in real-time [5]. Humans have one heart. Heart disease is a major health issue. Predicting heart disease is one of the most difficult and important subjects in medical study. It involves observing symptoms such as chest congestion, chest pain, cold sweats, shortness of breath, and blood pressure [6]. IoT sensor values are used to forecast heart disease diagnoses. In recent years, only a clinical and physical exam can diagnose disease.

A smartwatch can detect healthcare anomalies, such as irregular heartbeats in seniors. Healthcare monitoring equipment activates an alarm when it detects an irregular heart rhythm in a stationary person [7]. Medical applications use Bluetooth, RFID, and body area network. Clothing sensors measure blood pressure, ECG, and EMG. Smartwatches assess blood oxygen saturation, body temperature, the wearer's position, and movement. The observed information is sent to cloud storage and distributed to stakeholders such as family members, hospitals, and doctors. Wearable devices have gained popularity in healthcare monitoring systems, resulting in the Internet of medical things (IoMT) [8]. Data mining and ML save computing time and expenses. ML detects interventions and illnesses that change a patient's lifestyle. Usually, heart disease affects the elderly, although it's becoming more common across age groups [9]. Medical technology has progressed greatly in recent years. Rural areas have less healthcare. E-health reduces medical test costs, heart disease, and mortality. To identify and categorise HD in the context of e-healthcare, a successful FSHDL-HDDC method has been created. Since heart disease (HD) is a deadly condition, early detection of the condition is crucial to boosting patient survival rates. A significant issue in the analysis of clinical data is the earlier identification and classification of HD.

The key contribution of the manuscript.

- The new elite opposition-based squirrel search algorithm (EO-SSA) feature selection method is the core of the FSHDL-HDDC methodology.
- Additionally, the (ACNN-LSTM) model, an attention-based convolutional neural network (ACNN) with long short-term memory (LSTM), is used to identify HD by using clinical data.
- The development of the EO-SSA approach for the choice of appropriate parameters for HD detection and categorization serves as evidence of the study's uniqueness.
- Several simulations utilising benchmark HD datasets are executed, and the results are analysed from multiple perspectives to demonstrate that the FSHDL-HDDC technique yields superior results.

## 2. Literature Review

Khan and Algarni [10] proposed an IoMT architecture for the heart disease diagnosis with an ANFIS and modified salp swarm optimization (MSSO). The presented method enhances the search capacity with the Levy flight method. The learning parameter is enhanced by MSSO to provide effective outcomes for ANFIS. Haq et al. [11] presented a detection method with ML technique for diabetes diagnosis. The diabetes dataset, a medical dataset obtained from the patient's medical history, was used to test the proposed technique. They presented a filter model-based decision tree (Iterative Dichotomiser 3) method for the significant FS method. Also, the 3 ensemble learning models, Random Forest and AdaBoost, are employed for FS as well as compared the classification accuracy with wrapper-based FS models.

Meng et al. [12] included patients with stable ischemic heart disease (SIHD) in their study and applied a machine learning algorithm to patient-reported outcome (PRO) data gathered from activity trackers. Each participant was provided with a Fitbit Charge 2 for tracking their daily activities, and all the subjects have accomplished 8 PRO Measurement Information Systems. The 2 methods were constructed to categorise PRO scores with activity tracker data. Hashi and Zaman [13] compared and developed the performances of the conventional model with the presented method, which forecast heart disease by executing the LR, KNN, SVM, DT, and RF classification methods. The presented model assists in tuning the hyperparameter by means of the grid search model for the abovementioned 5 classification models.

Kishor and Jeberson [14] employed several ML techniques to produce information. An ML architecture has been presented for earlier diagnosis of heart disease, including IoT. The presented method is estimated by using KNN, DT, RF, MLP, NB, and L-SVM. Li et al. [15] developed accurate and efficient systems for the detection of heart diseases, and it depends on ML models. The proposed method depends on a classification algorithm that involves the KNN, SVM, DT, NB, ANN, and LR, whereas the typical FS method utilises minimal redundancy maximal relevance, relief, local learning to remove redundant and irrelevant features, and least absolute shrinkage selection operator. Also, presented a new fast conditional mutual data feature selection method for solving FS problems.

In Jain et al. [16], the 5 advanced ML methods (KNN, NB, LR, RF, and AdaBoost) have been used by the *Python* programming language for developing the predictive models. The efficiency was calculated on 30% of the information. Later, the predictive model was used in the cloud server for easy access through the internet. Zhang et al. [17] presented a heart disease predictive model which integrates DNN and embedded FS methods. In this strategy, the L1 norm and the linear SVC methodology are employed as a penalty to choose data subsets that are strongly associated with heart disease. This feature is fed into the DNN system. Rajaraman and Prakash [18] made a contribution to the development of an artificial classifier for patients suffering from congestive heart failure (CHF). This classifier differentiates between persons who are at low risk and those who are at high risk. In their study on the prevalence of heart disease, Kamalraj et al. [19] employed deep learning techniques, random forests, logistic regression, support vector machines (SVMs), hyperparameter tuning, and feature selection.

The accuracy performance of the HD classification system, created by Detrano et al. [20] utilising machine learning classification approaches, was 77%. The Cleveland dataset was analysed by using both the global evolutionary strategy and the features selection approach. In a different work, Gudadhe et al. [21] created a classification system for HD utilising multilayer perceptron and support vector machine (SVM) methods. With this approach, they were able to achieve an accuracy of 80.41 percent. A method for classifying high-definition (HD) photos by using a neural network and fuzzy logic was developed by Kahramanli and Allahverdi [22]. The accuracy of the classification system was found to be 87.4%.

A method for determining HD was developed by Kavitha et al. [23] based on a qualified medical diagnosis. The system was developed by using artificial neural networks, decision trees, and navigation bays as examples of machine learning prediction models. The accuracy of the NB classifier was 86.12%, that of the ANN classifier was 88.12%, and that of the DT classifier was 80.4%. Based on artificial neural network technology, Reshma et al. [24] established a three-phase technique for HD prediction in angina. With this approach, they were able to achieve an accuracy rate of 88.89%. A thorough medical decision support system for HD diagnosis was created by Samuel et al. The system makes use of a fuzzy analytical hierarchy technique along with a neural network. Performance accuracy was 91.10 percent for the suggested method.

## 3. The Proposed Model

**Figure 1:**
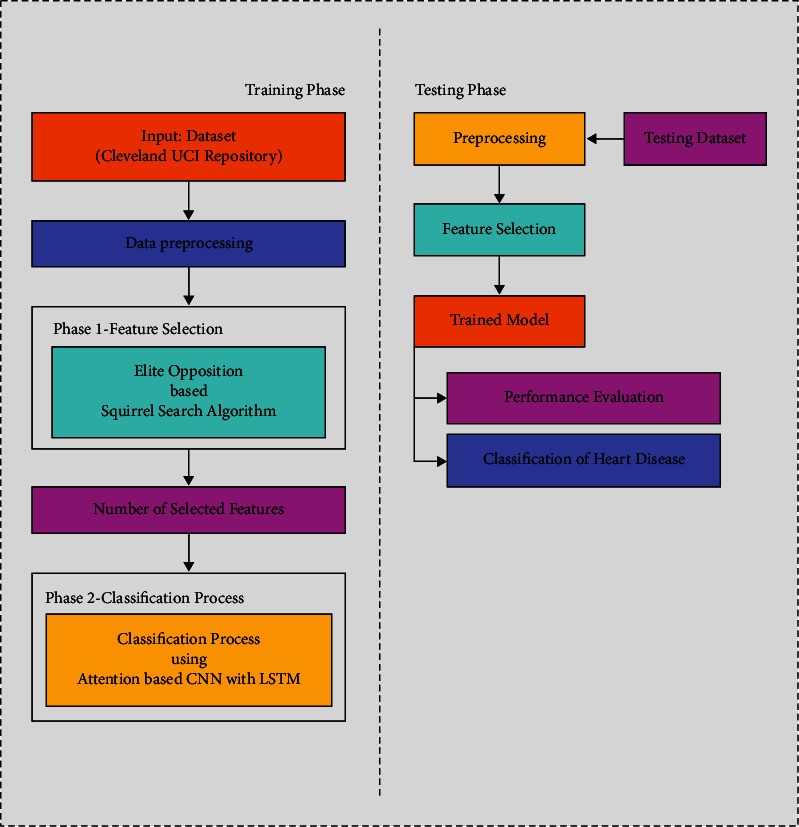
The overall process of the FSHDL-HDDC manner.

This study developed a successful FSHDL-HDDC strategy for recognising and categorising HD in the context of e-healthcare. Preprocessing (missing value replacement and data normalisation), the EO-SSA-based feature selection, and the ACNN-LSTM-based classification comprise the proposed FSHDL-HDDC approach. Using EO-SSA approaches to find the most relevant set of features improves classification accuracy while decreasing processing complexity. The FSHDL-HDDC technique procedure is depicted in Figure 1.

### 3.1. Data Pre-Processing

Initially, the medical data is preprocessed in two ways, namely missing value replacement and data normalisation. Initially, the data value is gathered from the UCI data set, where they are preprocessed that the replacement of missing data or requires noise removal. The noiseless data assist in efficient detecting patterns related to heart diseases. The median studentized residual method is used for removing noisy or undesirable data since it examines the correlations among the information in the datasets. This procedure of noise reduction improves the detection system of heart disease. This study developed an efficient FSHDL-HDDC technique for recognising and classifying HD within the context of e-healthcare. Replacement of missing values and data normalisation is the preprocessing step of the proposed FSHDL-HDDC technique, followed by feature selection based on the EO-SSA and classification based on the ACNN-LSTM. Using EO-SSA techniques to locate the most relevant set of features improves classification accuracy while reducing processing complexity. Figure 1 depicts the FSHDL-HDDC method in operation. The standardization method can be implemented by using various regression analyses and data distributions for the diagnosis of heart diseases.

### 3.2. Algorithmic Process Involved in the EO-SSA Based Feature Selection Technique

Once the healthcare data is preprocessed, the EO-SSA technique is utilized to pick an optimal subset of features. The typical SSA upgrades the locations of individuals as per the type of individuals, the current season, and whether a predator appears [19]. Assume that *N* number of the population, *FS*_*u*_ and *FS*_*L*_ represent the upper and lower limits of the searching space, respectively. *N* individuals are arbitrarily formed as follows:

(1) $$\begin{array}{c} FS_{i}=FS_{L}+ran\;d\left(1,D\right)\times \left(FSu-FS_{L}\right). \end{array}$$

*FS*_*i*_ denotes the ith individual, (*i*=1 … *N*); rand indicates an arbitrary value within [0, 1], and *D* signifies the dimensionality of the problem. The individual updates their position by gliding to the acorn trees or hickory trees.

(2) $$\begin{array}{c} \left\{\begin{array}{cc} FS_{i}^{t+1}=FS_{i}^{t}+d_{g}\times G_{c}\times \left(F_{h}^{t}-FS_{i}^{t}\right) & if\;r>P_{dp},\\ \text{randomlocation} & or\;else, \end{array}\right. \end{array}$$

(3) $$\begin{array}{c} \left\{\begin{array}{cc} FS_{i}^{t+1}=FS_{i}^{i}+d_{g}\times G_{c}\times \left(F_{ai}^{i}-FS_{i}^{i}\right) & if\;r>P_{dp},\\ \text{randomlocation} & or\;elese, \end{array}\right. \end{array}$$

*r* indicates an arbitrary value within [0,1], *P*_*dp*_ values at 0.1 denote the predator appearance possibility, When *r* > *P*_*dp*_, no predators appear, the squirrel glides in the forest to find food, and the individual is safe when *r* ≤ *P*_*dp*_, the predator appears. The squirrel is forced to narrow the scope of activities, the individual is threatened, and the position is randomly relocated, *t* signifies the existing iteration, *G*_*c*_ implies the constant with the values of 1.9, and *F*_*ai*_(*i*=1,2,…*N*_*fs*_) means the individual arbitrarily elected from *F*_*a*_,*dg* representing the gliding distance as follows:

(4) $$\begin{array}{c} d_{g}=\frac{h_{g}}{\tan\left(\varphi \right)\times sf}, \end{array}$$

*hg* indicates the constant value 8, *sf* denotes the constant value 18, and tan(*φ*) signifies the gliding angle as follows [20]:

(5) $$\begin{array}{c} \tan\left(\varphi \right)=\frac{D}{L}. \end{array}$$

*D* denotes the drag force and *L* represent the lift force as follows:

(6) $$\begin{array}{c} D=\frac{1}{2\rho V^{2}SC_{D}}, \end{array}$$

(7) $$\begin{array}{c} L=\frac{1}{2\rho V^{2}SC_{L}}, \end{array}$$

**Figure 2:**
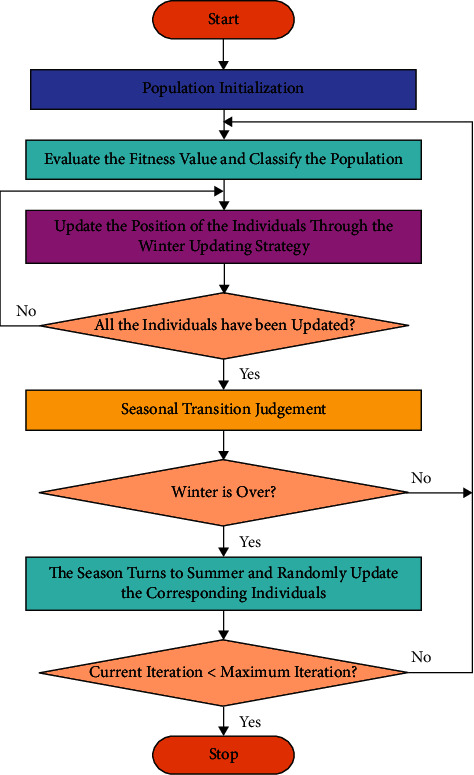
Flowchart of SSA.

*ρ*,*V*,*S*, and *C*_*D*_ denotes constant that is equivalent to 1.204 kg *m*^−3^, 5.25*ms*^−1^, 154*cm*^2^, and 0.6, correspondingly; *C*_*L*_ denotes an arbitrary value in the range 0.675 –1.5. In the initial stage of all the iterations, the typical SSA needs that the entire population is in winter. Once each individual has been upgraded, either the seasonal variations are judged as follows.  Figure 2 depicts the flowchart of SSA.

(8) $$\begin{array}{c} S_{c}^{t}=\sqrt{\overset{D}{\underset{k=1}{\sum }}{\left(F_{ai,k}^{t}-F_{h,k}^{t}\right)}^{2}}\;i=1,2,\ldots ,N_{fs}, \end{array}$$

(9) $$\begin{array}{c} S_{\min\;}=\frac{10e^{-6}}{{\left(365\right)}^{i/\left(T/2.5\right)}}. \end{array}$$

*T* denotes the maximal amount of iterations, if *S*_*c*_^*t*^ < *S*_min  /_. If winter has ended, the season turns to summer; otherwise, the season remains the same. Once the season turns into summer, each individual who glides to *F*_*h*_ stays at the upgraded position, and each individual who glides to *F*_*a*_ and does not meet with predators relocates their locations as follows:

(10) $$\begin{array}{c} FS_{inew}^{i+1}=FS_{L}+Le^{\prime }vy\left(n\right)\times \left(FSu-FS_{L}\right). \end{array}$$

For nonnegative random variables, the Levy distribution is a probability distribution that is stable and continuous. Levy represents the arbitrary walk whose step follows the Levy distribution as

(11) $$\begin{array}{c} Le^{\prime }vy\left(x\right)=0.01\times \frac{r_{a}\times \sigma }{\left|r_{b}{|}^{1/\beta }\right.}. \end{array}$$

*β* indicates the constant value 1.5, *σ* as

(12) $$\begin{array}{c} \sigma ={\left(\frac{\Gamma \left(1+\beta \right)\times \sin\left(\pi \beta /2\right)}{\Gamma \left(1+\beta /2\right)\times \beta \times 2^{\left(\beta -1/2\right)}}\right)}^{\frac{1}{\beta }}. \end{array}$$

The optimization procedure of the SSA is considered as the continuous transformation of their searching space. Once the method falls as to local optimal, the searching space is complex for having the optimum solution globally. It is proposed to enhance the global search capabilities of the SSA by using the elite opposition-based learning (EOBL) technique (also known as its capacity for exploration). Because the opposition solution of generated EOBL may not be beneficial when compared to the existing search space to seek the global optimum solution, therefore, utilize the EOBL approach.

EOBL is a modern approach in the area of intelligence computation: Assume the elite individual from the existing population is*X*_*e*_=(*x*_*e*,1_, *x*_*e*,2_,…, *x*_*e*,*D*_), for an individual *X*_*i*_=(*x*_*i*,1_, *x*_*i*,2_,…, *x*_*i*,*D*_), and the elite opposition solution ${\tilde{X}}_{i}=\left({\tilde{x}}_{i,1},{\tilde{x}}_{i,2},\ldots ,{\tilde{x}}_{i,D}\right)$ of *X*_*i*_ is determined by:

(13) $$\begin{array}{c} \bar{x}=\eta *\left(da_{j}+db_{j}\right)-x_{e,j}. \end{array}$$

where as *i*=1, 2,…, *NP*,*NP* represent the population size, *j*=1,2,…, *D*, *η* ∈ *U*(*O*, 1), *η* indicates a generalized coefficient, and [*da*_*j*_, *db*_*j*_] denotes the dynamic boundary of the *jth* dimension searching space as follows [21]:

(14) $$\begin{array}{c} da_{j}=\min\left(x_{i,j}\right), \end{array}$$

(15) $$\begin{array}{c} db_{j}=\max\left(x_{i,j}\right)\text{.} \end{array}$$

Furthermore, when the operator of the dynamic boundary makes ${\tilde{x}}_{i,j}$ jumps out of [*da*_*j*_, *db*_*j*_], the subsequent process is utilized for resetting ${\tilde{x}}_{i,j}$:

(16) $$\begin{array}{c} {\tilde{x}}_{i,j}=ran\;d\left(da_{j},db_{j}\right). \end{array}$$

The elite opposition-based squirrel search algorithm (EO-SSA) is created by using the FSHDL-HDDC technology to find the ideal subset of attributes. An attention-based convolutional neural network (ACNN-LSTM), also known as a CNN with long short-term memory, is used to analyse medical data to find occurrences of HD (LSTM). The EOBL evaluates both the elite population and the present population while concurrently creating the opposition's population based on the elite individual. Additionally, it fully makes use of the characteristics of the exceptional individual to produce search data that is more effective than that produced by the average person, somewhat increasing population variety. EOBL might improve SSA's capacity for foreign travel.

When the feature vector size is N, the number of distinct feature combinations tends to rise to 2N during the feature selection process with the EO-SSA, which is a large space for a thorough search. The proposed hybrid model is utilized to dynamically scan the feature space and precisely integrate features. In order to create an optimal outcome that reduces the number of subsets of selected features while boosting output accuracy for a given classification, feature selection must satisfy one or more criteria [22]. As previously mentioned, the fitness function that was employed to determine the solution in this case allowed for the achievement of the following harmony between the two goals.

(17) $$\begin{array}{c} \text{fitness}=\alpha {∆}_{R}\left(D\right)+\beta \frac{\left|Y\right|}{\left|T\right|}. \end{array}$$

In which ΔR (*D*) represents the classifier error rate. |*Y* | indicates the size of the subset, and |*T*| signifies the overall amount of the feature comprised in the existing data set. *α* denotes a ∈ [0, 1] parameter associated with the relative weight of the classification error rate, similarly and *β* = 1 − *α* means the importance of the reduction feature. Priority and weight have been allocated in place of counting the number of features that were categorised as successful. Solutions with comparable classification accuracy but fewer selected features are disregarded when just classification accuracy is taken into account by the estimate function, which is a critical step in reducing the dimensionality issue. This is true since the estimation function only takes classification accuracy into account.

### 3.3. Process Involved in the ACNN-LSTM Based Classification

Next to the optimal selection of features, the classification process is carried out by the utilization of the ACNN-LSTM model. In this study, the hybrid integration of LSTM and CNN methods has been employed. In order to process the consecutive data, RNN is extensively utilized. The current input and the past output are concatenated with this RNN. By presenting multiple gates, LSTM problems could be solved easily. The LSTM gate construction can be used to randomly memorise the input [23]. The least important knowledge is fully lost in tandem with the most crucial knowledge. As a result, an exact computation of the amount of data that can be preserved in the current state has been produced. The sigmoid function is used as an input, producing a value between zero and one and so selecting the currently retained data. Its inputs are the current input of a function *X t* and the output from the previous state *h* (*t*-1). The input and forget gates, which are also used to start the next state's C *t* complete state from the output of the current unit, are utilized to obtain the next state's *h t* hidden layer. The amount of cell state established for projecting an output is indicated by the value of *t* that the sigmoid function returns, which ranges from zero to one. Once the multiplication of cell state data arises with *o*_*t*_, it has been activated by using tan *h* layer.Therefore, the output detail of the LSTM determination *h*_*t*_ are modelled. For the LSTM, the respective relations among the multiple gates are arithmetically formulated by the equation:

(18) $$\begin{array}{c} z_{t}=\tanh\left(W_{z}\left[h_{t-1},X_{t}\right]+b_{z}\right)\text{,} \end{array}$$

(19) $$\begin{array}{c} i_{t}=sigmoi\;d\left(W_{i}\left[h_{t-1},X_{t}\right]+b_{i}\right)\text{,} \end{array}$$

(20) $$\begin{array}{c} f_{t}=sigmoi\;d\left(W_{f}\left[h_{t-1},X_{t}\right]+b_{f}\right)\text{,} \end{array}$$

(21) $$\begin{array}{c} o_{t}=sigmoi\;d\left(W_{o}\left[h_{t-1},X_{t}\right]+b_{o}\right)\text{,} \end{array}$$

(22) $$\begin{array}{c} c_{t}=f_{t}\cdot C_{t-1}+i_{t}\cdot z_{t}\text{,} \end{array}$$

(23) $$\begin{array}{c} h_{t}=o_{t}\cdot \tanh\left(c_{t}\right)\text{.} \end{array}$$

It becomes harder to understand the data from a long-term perspective as the length of the input sequence increases due to the gradient problem explosion. The forget gate explains how to ignore cell state data, whereas the input gate explains how to calculate novel input. A depiction of the outcome value can be produced based on the cell's current state and contents. The CNN-LSTM methodology offers four channels for continuously inputting a large number of embedding labels to achieve varied feature attributes.

(24) $$\begin{array}{c} c=con\nu \left(X,K\right)+b\text{,} \end{array}$$

*x* is a symbol that can be used to express the length of the sequence. LSTM is an abbreviation for the combined LSTM process (*x*). The CNN and LSTM-NN both use parallel and series architectures to accomplish their respective goals. The RNN is applied widely so that the consecutive data can be processed. This RNN joins the most recent input with the most recent output from the previous iteration. Problems with the LSTM could be readily handled by showing a network of several gates. In general, the series structure is widely utilized despite the data loss because of the nature of the convolutional method. Now, series structures are substituted with parallel structures and attain an effective outcome. In all the channels, the recording of the structure can be made as follows:

(25) $$\begin{array}{c} \text{channel}\left(x\right)=\left[con\nu \left(x\right)⊕LSTM\left(x\right)\right]\text{.} \end{array}$$

With *x* represent the input and the output, formulated as *C*_*out*_ and*W*_*out*_:

(26) $$\begin{array}{c} C_{out}=\text{channe}l_{\text{embedding}}=\nu_{w}\left(x\right)\text{,} \end{array}$$

(27) $$\begin{array}{c} W_{out}=\text{channe}l_{\text{embedding}}=\nu_{c}\left(x\right)\text{.} \end{array}$$

The outcome and interpretation of the output of the 4-channel mechanism are integrated as a hidden layer output

(28) $$\begin{array}{c} h=\left[C_{out}⊕W_{out}\right]\text{.} \end{array}$$

To FC layer, these hidden layer results are transmitted, and lastly, to classifier output, the Softmax layer is employed as follows:

(29) $$\begin{array}{c} \hat{y}=\text{softmax}\left(\text{dense}\left(h\right)\right)\text{.} \end{array}$$

Following is a description of the representations by using all four channels. The *w* weight score, which is an important part of the dynamic flexible weight structures, is denoted as follows:

(30) $$\begin{array}{c} e_{i}=\nu_{a}^{T}\tanh\;\left(W_{r}h_{i}+b\right)\text{,} \end{array}$$

(31) $$\begin{array}{c} h_{i}=\langle h_{t}^{\prime }:c_{t}\rangle \text{.} \end{array}$$

In which *h*_*t*_′ represent the LSTM outcome at a certain time *t*,*h*_*i*_ represent the hidden layer outcome, *c*_*t*_ denotes the state in LSTM, *ν*_*a*_ signifies the arbitrary initiation vector, *b* denotes the bias, i.e., arbitrarily initiated, and *W*_*r*_ implies the arbitrary initiation weight matrix.

(32) $$\begin{array}{c} w=\frac{\exp\left(e_{i}\right)}{\varepsilon }\left[\overset{T_{x}}{\underset{k=1}{\sum }}\exp\left(e_{i}k\right)\right]\text{.} \end{array}$$

While the sequence length can be represented by *x*. The dynamic adoptive weight has weighted to the resultant vector *c*_*i*_ as

(33) $$\begin{array}{c} c_{i}=\overset{T_{x}}{\underset{j=1}{\sum }}w\cdot h_{j}\text{.} \end{array}$$

## 4. Experimental Validation

By using the Cleveland HD benchmark dataset, the experimental results of the FSHDL-HDDC approach are assessed [24–27]. The dataset consists of 303 samples, each of which has two classes and 13 features (age, sex, cp, restbps, chol, fbs, restecg, thalach, examg, and oldpeak). The findings are assessed over ten different rounds.  Table 1 contrasts the provided EOSSA-FS features with those of competing FS strategies and shows the ideal cost. The findings show that the cost of 0.4260 and seven features of the EOSSA-FS method's successful FS results were delivered.

**Figure 3:**
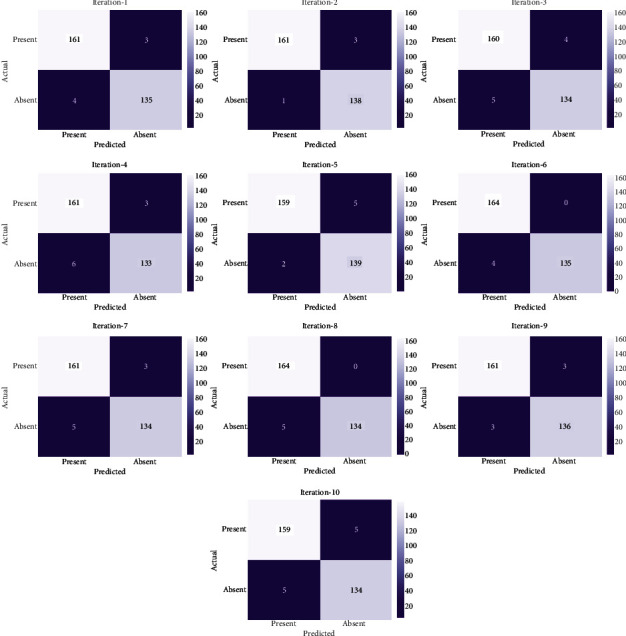
Confusion matrix of the FSHDL-HDDC technique with different runs.

The FSHDL-HDDC technique was applied to the categorization of HD ten times, which resulted in the production of ten confusion matrices, which are depicted in Figure 3. With iteration-1, the FSHDL-HDDC technique has classified 161 instances into “presence of HD” and 135 instances into “absence of HD.” Besides, with iteration-4, the FSHDL-HDDC manner has classified 161 instances into “presence of HD” and 133 instances into “absence of HD.” Along with that, with iteration-6, the FSHDL-HDDC method has classified 164 instances into “presence of HD” and 135 instances into “absence of HD.” Likewise, with iteration-8, the FSHDL-HDDC technique has classified 164 instances into “presence of HD” and 134 instances into “absence of HD.” Lastly, with iteration-10, the FSHDL-HDDC method has classified 159 instances into “presence of HD” and 134 instances into “absence of HD.”

A thorough analysis of the HD classification outcomes produced by using the FSHDL-HDDC technique after 10 iterations can be seen in Table 2.

**Figure 4:**
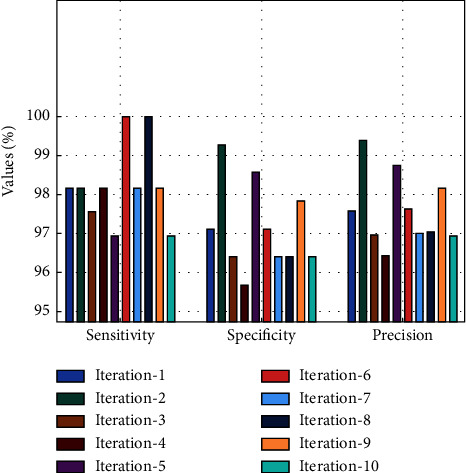
*Sen*_*y*_, *Spec*_*y*_, and *Prec*_*n*_ analysis of the FSHDL-HDDC approach.

Figure 4 depicts the global sensitivity, specificity, and precision analysis of the FSHDL-HDDC method after 10 iterations. The results revealed that the FSHDL-HDDC algorithm had reached improved outcomes under every iteration [28–32]. For instance, with iteration-1, the FSHDL-HDDC method has offered *sens*_*y*_, *spec*_*y*_, and *prec*_*n*_ of 98.17%, 97.12%, and 97.58%, respectively. Along with that, with iteration-5, the FSHDL-HDDC methodology has obtainable *sens*_*y*_, *spec*_*y*_, and *prec*_*n*_ of 96.95%, 98.58%, and 98.76%, correspondingly. Moreover, with iteration-10, the FSHDL-HDDC system has existing *sens*_*y*_, *spec*_*y*_, and *prec*_*n*_ of 96.95%, 96.40%, and 96.95%, correspondingly.

**Figure 5:**
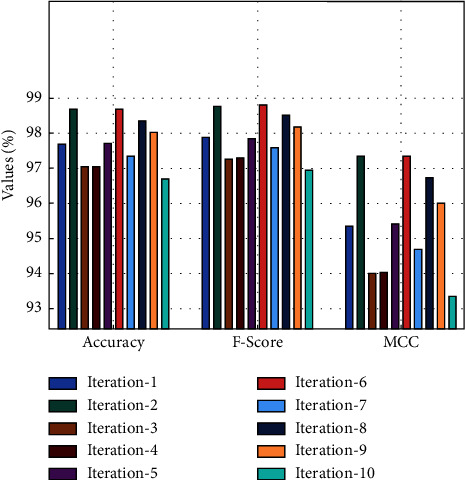
*Acc*_*y*_, *F*_*score*_, and *MCC* analysis of the FSHDL-HDDC approach.

Figure 5 gives the overall *acc*_*y*_, *F*_*score*_, and *MCC* analysis of the FSHDL-HDDC manner in 10 iterations. The outcomes exposed that the FSHDL-HDDC approach has gained increased outcomes under every iteration [33–35]. For instance, with iteration-1, the FSHDL-HDDC manner has obtainable *acc*_*y*_, *F*_*score*_, and *MCC* of 97.69%, 97.87%, and 95.35%, correspondingly. Besides, with iteration-5, the FSHDL-HDDC algorithm has presented *acc*_*y*_, *F*_*score*_, and *MCC* of 97.70%, 97.85%, and 95.41%, correspondingly. Furthermore, with iteration-10, the FSHDL-HDDC manner has offered *acc*_*y*_, *F*_*score*_, and *MCC* of 96.70%, 96.95%, and 93.35%, correspondingly.

**Figure 6:**
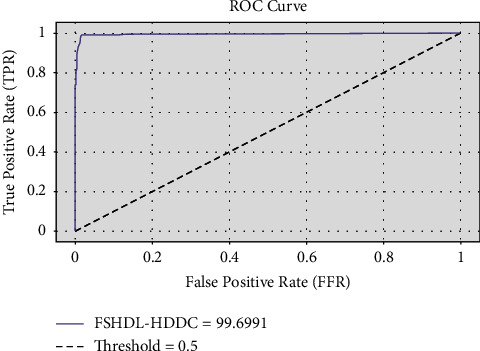
ROC analysis of the FSHDL-HDDC technique.

Figure 6 shows a clear ROC analysis of the FSHDL-HDDC technique on the test dataset. The FSHDL-HDDC technique can offer superior performance with a higher ROC of 99.6991, according to the data.

**Figure 7:**
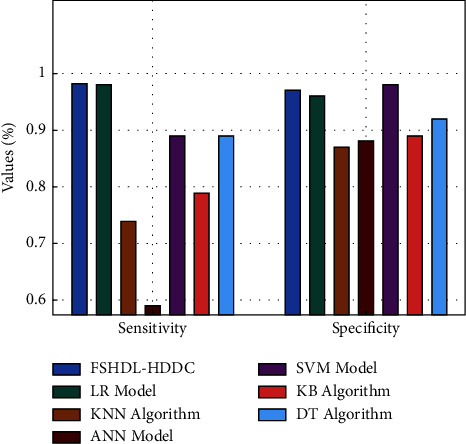
Comparative analysis of the FSHDL-HDDC technique in terms of *Sen*_*y*_ and *Spec*_*y*_.

A thorough comparison of the FSHDL-HDDC technology and alternative approaches is provided in Table 3. Based on current practises, Figure 7 presents a succinct sensory and structural analysis of the FSHDL-HDDC system. The data showed that the NB, KNN, and ANN models failed to give accurate HD classification results when sens *y* and spec *y* had lower values. Furthermore, the performance of the DT and LR models has been slightly enhanced with moderate sens *y* and spec *y* values. Despite the SVM model's efforts to produce outcomes that were close to ideal, the proposed FSHDL-HDDC strategy outperformed the other approaches, with maximum sens *y* and spec *y* values of 0.9712 and 0.9823, respectively.

**Figure 8:**
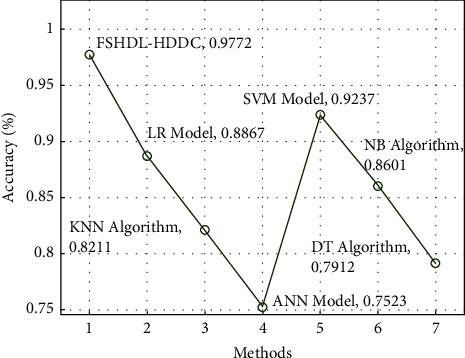
Accuracy analysis of the FSHDL-HDDC approach with existing manner.

Figure 8 depicts a comparison between the FSHDL-HDDC methodology and more contemporary techniques. With lower values of acc *y*, the DT, KNN, and ANN classification models failed to produce useful HD classification results [36]. After that, with modest values of acc *y*, the performance of the NB and LR techniques is somewhat enhanced. However, the FSHDL-HDDC strategy demonstrated the other ways with the greatest acc *y* of 0.9772, although the SVM methodology aimed to obtain nearly ideal results.

**Figure 9:**
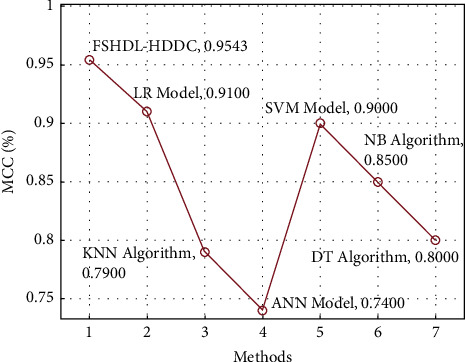
MCC analysis of the FSHDL-HDDC manner with existing algorithm.

Figure 9 showcases a detailed MCC analysis of the FSHDL-HDDC approach with recent manners. The outcomes outperformed the DT, KNN, and ANN manners have attained ineffective HD classification outcomes with minimal values of MCC.

Along with that, the NB and SVM techniques have achieved slightly enhanced performance with moderate values of MCC. Similarly, the LR technique has tried to accomplish near optimum outcomes. The proposed FSHDL-HDDC methodology has demonstrated other manners with a higher MCC of 0.9543.

**Figure 10:**
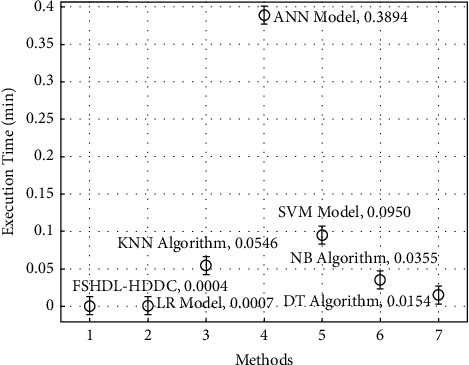
Execution time analysis of the FSHDL-HDDC approach with existing manners.

The execution time analysis of the FSHDL-HDDC technique is shown in Figure 10 lastly. The graph shows that the ANN approach has produced poor results with an elevated ET of 0.3894 seconds. The ETs obtained by applying the SVM, KNN, and NB models were slightly lower, at 0.0950 min, 0.0546 min, and 0.0355 min, respectively. The LR and DT models have made attempts to reach ETs of 0.0007 min and 0.0154 min, respectively, in the interim. The FSHDL-HDDC strategy presented has surpassed the prior techniques with a decreased ET of 0.0004 min. The tables and graphs above make it abundantly evident that the FSHDL-HDDC approach can identify and classify HD in the context of e-healthcare.

## 5. Conclusion

An effective FSHDL-HDDC technique has been developed in this study to recognise and classify HD in the context of e-healthcare. The proposed FSHDL-HDDC technique encompasses preprocessing (missing value replacement and data normalisation), EO-SSA-based feature selection, and ACNN-LSTM-based classification. Therefore, the main problems with these previous methods are their poor accuracy and lengthy computation times, which may be caused by the inclusion of unnecessary features in the dataset. To detect HD properly, new methods are required in order to address these issues. The design of the EO-SSA technique helps to properly choose the useful set of features, thereby improving the classification accuracy and reducing the computational complexity. To show off the improved performance of the FSHDL-HDDC technique, a number of simulations using benchmark HD datasets are run, and the results are then thoroughly examined. The FSHDL-HDDC technology is better than other current techniques, according to several simulation results. In the future, the ACNN-LSTM approach's hyperparameters will be optimised by using metaheuristic techniques. The basis for metaheuristic algorithms could be either demographic data or natural cues because metaheuristics optimise real issues. The use of Iterated Local Search, Hill Climbing, Genetic Algorithms, Simulated Annealing, Tabu Search, and Ant Colony Optimization can be used to address complex scheduling, space allocation, and clustering problems.

## Figures and Tables

**Table 1:** FS analysis of the EOSSA-FS technique.

Algorithm	Selected feature	Best cost
Relief	13, 9, 3, 11	0.4564
MRMR	12, 8, 3, 5, 11, 12, 2, 13	0.4541
LASSO	2, 12, 9, 3, 11, 13	0.4538
LLBFS	13, 12, 3, 2, 11, 10	0.4517
FCMIM	2, 3, 4, 5, 7, 8, 9, 10, 11, 13	0.4436
EOSSA-FS	6, 7, 2, 3, 4, 11, 13	0.4260

**Table 2:** Result analysis of the FSHDL-HDDC technique with different measures.

No. Of iterations	Sensitivity	Specificity	Precision	Accuracy	*F*-score	MCC
Iteration-1	98.17	97.12	97.58	97.69	97.87	95.35
Iteration-2	98.17	99.28	99.38	98.68	98.77	97.35
Iteration-3	97.56	96.40	96.97	97.03	97.26	94.02
Iteration-4	98.17	95.68	96.41	97.03	97.28	94.03
Iteration-5	96.95	98.58	98.76	97.70	97.85	95.41
Iteration-6	100.00	97.12	97.62	98.68	98.80	97.37
Iteration-7	98.17	96.40	96.99	97.36	97.58	94.69
Iteration-8	100.00	96.40	97.04	98.35	98.50	96.72
Iteration-9	98.17	97.84	98.17	98.02	98.17	96.01
Iteration-10	96.95	96.40	96.95	96.70	96.95	93.35
Average	98.23	97.12	97.59	97.72	97.90	95.43

**Table 3:** Comparative analysis of the FSHDL-HDDC method with existing manners.

Methods	Accuracy	Specificity	Sensitivity	MCC	Execution time (min)
FSHDL-HDDC	0.9772	0.9712	0.9823	0.9543	0.0004
LR model	0.8867	0.9600	0.9800	0.9100	0.0007
KNN algorithm	0.8211	0.8700	0.7400	0.7900	0.0546
ANN model	0.7523	0.8800	0.5900	0.7400	0.3894
SVM model	0.9237	0.9800	0.8900	0.9000	0.0950
NB algorithm	0.8601	0.8900	0.7900	0.8500	0.0355
DT algorithm	0.7912	0.9200	0.8900	0.8000	0.0154

## Data Availability

The data used to support the findings of the study can be obtained from the corresponding author upon request.
